# Flexible Quasi-Solid-State Composite Electrolyte of Poly (Propylene Glycol)-co-Pentaerythritol Triacry-Late/Li_1.5_Al_0.5_Ge_1.5_(PO_4_)_3_ for High-Performance Lithium-Sulfur Battery

**DOI:** 10.3390/ma14081979

**Published:** 2021-04-15

**Authors:** Zekun Deng, Zhenyang Zheng, Wenhong Ruan, Mingqiu Zhang

**Affiliations:** 1 Key Laboratory for Polymeric Composite and Functional Materials of Ministry of Education, School of Chemistry, Sun Yat-sen University, Guangzhou 510275, China; dengzk1996@163.com (Z.D.); zhengzhy8@mail2.sysu.edu.cn (Z.Z.); ceszmq@mail.sysu.edu.cn (M.Z.); 2 Guangdong Provincial Key Laboratory for High Performance Polymer-Based Composites, Guangzhou 510275, China

**Keywords:** lithium sulfur battery, quasi-solid-state composite electrolyte, polysulfides shuttle effect, Li-ion conductivity, cycling performance

## Abstract

With a higher theoretical specific capacity (1675 mAh g^−1^) and energy density (2600 Wh kg^−1^), the lithium-sulfur (Li-S) battery is considered as a promising candidate for a next-generation energy storage device. However, the shuttle effect of polysulfides as well as the large interfacial impedance between brittle solid electrolyte and electrodes lead to the capacity of the Li-S battery decaying rapidly, which limits the practical commercial applications of the Li-S battery. Herein, we reported a facile in situ ultraviolet (UV) curing method to prepare a flexible quasi-solid-state composite electrolyte (QSSCE) of poly(propylene glycol)-co-pentaerythritol triacrylate/Li_1.5_Al_0.5_Ge_1.5_(PO_4_)_3_ (PPG-co-PETA/LAGP). By combining the high Li-ion conductivity and mechanical strength of inorganic NASICON-structure LAGP and good flexibility of the crosslinked PPG-co-PETA with nanopore structure, the flexible QSSCE with 66.85 wt% LAGP exhibited high Li-ion conductivity of 5.95 × 10^−3^ S cm^−1^ at 25 °C, Li-ion transference number of 0.83 and wide electrochemical window of ~5.0 V (vs. Li/Li^+^). In addition, the application of QSSCE in the Li-S battery could suppress the shuttle effect of polysulfides effectively, thus the Li-S battery possessed the excellent electrochemical cyclic performance, showing the first-cycle discharge-specific capacity of 1508.1 mAh g^−1^, the capacity retention of 73.6% after 200 cycles with 0.25 C at 25 °C and good rate performance.

## 1. Introduction

In order to alleviate the problems of energy shortage and environmental pollution related to fossil fuels, there is an urgency to develop economic and efficient energy storage technologies based on renewable resources [1,2,3]. Among them, the lithium-sulfur (Li-S) battery, with high theoretical specific capacity (1675 mAh g^−1^) and energy density (2600 Wh kg^−1^), which uses environmentally friendly, low-cost and abundant storage of sulfur as an active substance, is considered as the most promising next generation of electrochemical energy storage system [4,5]. However, the major obstacle limiting the commercial application of Li-S batteries is the so-called “shuttle effect”, which occurs in the electrochemical process of the Li-S battery. During the discharge process, the element sulfur (S_8_) is first reduced to form polysulfide (Li_2_S_x_, 4 ≤ x ≤ 8), then the intermediate polysulfides dissolve into organic solvent of the liquid electrolyte and migrate to the anode side, and then are reduced to insoluble solid Li_2_S_2_ and Li_2_S depositing on the Li anode [6,7]. The shuttle effect results in low utilization of active material, fast capacity decay and destruction of lithium metal anode [8,9,10].

In order to solve the problem of the shuttle effect, using all-solid-state electrolytes (ASSE) instead of liquid electrolyte is considered as one of the most effective strategies [11,12]. Owing to the elimination of volatile organic solvents, ASSE can eliminate the dissolution and migration of polysulfides completely [13,14,15]. Inorganic solid electrolyte (ISE), including NASICON-type Li_1+x_Al_x_Ti_2-x_(PO_4_)_3_ (LATP) [16] and Li_1+x_Al_x_Ge_2-x_(PO_4_)_3_ (LAGP) [17], garnet-type Li_7_La_3_Zr_2_O_12_ (LLZO) [18], perovskite-type Li_3x_La_(2/3)-x_TiO_3_ (LLTO) [19] and sulfide-based xLi_2_S·(1-x)P_2_S_5_ [20], have been developed for all-solid-state Li-S batteries in recent years, due to their high Li-ion conductivity (10^−4^ S cm^−1^) and ion transference number (close to 1) at room temperature, and wide electrochemical window up to 6.0 V (vs. Li/Li^+^) [21,22,23]. However, the brittleness of ISE and large interfacial resistance between ISE and electrodes restrict the practical application of ISE in Li-S batteries [24,25].

Quasi-solid-state electrolyte (QSSE), another type of electrolyte by adding a small amount of the liquid electrolyte into the ISE, can solve the problem of large interfacial resistance mentioned above, to some extent [26,27]. For making QSSE, a solid electrolyte such as LAGP ceramic powder was first pressed into thick sheets and dropped in liquid electrolyte or was prepared into mixed conductive slurry coated on the surface of the sulfur electrode [28,29,30]. Inorganic solids can suppress the diffusion of polysulfides from the sulfur cathode side by physical blocking, and a liquid electrolyte makes solid electrolytes and electrodes wet, so the QSSE combines the advantages of both solid electrolytes and liquid electrolytes. However, there are still some problems with using QSSE for Li-S batteries, including poor liquid electrolytes uptake and inherent brittleness of the inorganic solid layer, which make the electrochemical performance of the Li-S battery still not stable and decay fast.

A sandwich structure by introducing a polymer interlayer between LAGP QSSE and electrodes is an alternative way [31,32,33]. The polymer interlayer can provide good interfacial compatibility as well as liquid electrolyte holdup to reduce interfacial impedance. However, there is no change in the fragileness of ceramic sheets at all, so it is still difficult to be used in a flexible device [34,35]. To keep in pace with these rapid developments, high-performance devices with excellent mechanical flexibility are essentially required. A feasible method to solve these issues is blending polymer with the QSSE electrolyte, obtaining a quasi-solid-state composite electrolyte (QSSCE). However, the challenge is that polymer matrix such as PEO, PVDF and PAA used for blending cannot block the diffusion of the polysulfides effectively. Therefore, developing a novel polymer matrix that can suppress the shuttle effect is a hopeful route to develop polymer/inorganic powder blended QSSCE.

The poly(propylene glycol)-co-pentaerythritol triacrylate (PPG-co-PETA) is a crosslinked polymer with nanopore structure, reported by our group previously [36]. The PPG-co-PETA used as a separator in the Li-S battery could effectively block the shuttle effect due to its micropore structure, the size of which is smaller than the particle size of polysulfides. However, the disadvantages of PPG-co-PETA are low ionic conductivity and low mechanical strength. Blending PPG-co-PETA with inorganic powder to obtain QSSCE might combine the advantages of both ISE and microporous polymer to solve the above-mentioned problems. On the one hand, inorganic powder could provide high Li-ion conductivity and sufficient mechanical properties for QSSCE; on the other hand, PPG-co-PETA not only could solve the problems of ISE in brittleness and large interfacial impedance with electrodes, but also could absorb the liquid electrolytes due to its porous structure. In addition, such QSSCE could inhibit the shuttle effect of polysulfides more effectively due to the synergism of the micropore structure of PPG-co-PETA and the physical blocking of ISE.

Herein, a QSSCE of poly(propylene glycol)-co-pentaerythritol triacrylate/Li_1.5_Al_0.5_Ge_1.5_(PO_4_)_3_ (PPG-co-PETA/LAGP) was prepared by the in situ UV curing method, which is an efficient and environmentally friendly method. In this study, the monomer and additives for preparing polymer were first mixed with LAGP powder, then the reactants were cured under UV irradiation for a short time and PPG-co-PETA was formed in ceramic powder, and the composite was pressed into a flexible layer to prepare QSSCE. The structure, morphology and electrochemical performances of flexible QSSCE were evaluated and used to assemble the Li-S battery. Moreover, the mechanism of QSSCE in suppressing the shuttle effect of polysulfides was explored. It is hoped that the research on the novel QSSCE can accelerate the development of Li-S batteries, especially for flexible devices.

## 2. Materials and Methods

### 2.1. Synthesis of LAGP Powder

Lithium carbonate (Li_2_CO_3_), aluminum oxide (Al_2_O_3_), germanium oxide (GeO_2_) and ammonium dihydrogen phosphate (NH_4_H_2_PO_4_) were well-mixed on a ball mill for 1 h, using stoichiometric ratios. The mixture was firstly heated at 180 °C for 4 h in air at a rate of 3 °C min^−1^ and then heated at 700 °C for 4 h in air at the same rate to break down NH_4_H_2_PO_4_ and Li_2_CO_3_ completely. The mixture was subsequently heated at 950 °C for 10 h in air. The temperature was raised slowly at a rate of 1 °C min^−1^ and cooled at a rate of 3 °C min^−1^. The as-synthesized LAGP powder was further milled on a ball mill for 24 h.

### 2.2. Synthesis of PPG-co-PETA/LAGP Composites

The ratios of all reactants are shown in Supplementary Table S1. A certain amount of pentaerythritol triacrylate (PETA) as a cross-linking agent and 2,2-Dimethoxy-2-phenylacetophenone (DMPA) as a photo-initiator were firstly dispersed into propylene oxide (PO). A homogeneous suspension was obtained by adding LAGP powder into the above-mentioned solution mixture following magnetic stirring and ultrasonic dispersion. The suspension was irradiated under UV light (λ = 254 and 365 nm, YZ-UV12, Yan Zheng Instrument, Shanghai, China) for 1 h at room temperature. The raw product was purified in a Soxhlet extractor with acetone at 65 °C for 24 h and dried in a vacuum oven at 80 °C for 24 h. The actual contents of LAGP were measured by thermogravimetric analysis (TGA, TGA-Q50, TA Instrument, USA).

### 2.3. Fabrication of PPG-co-PETA/LAGP Composite Membranes

In a small amount of alcohol and 0.015 g polytetrafluoroethylene (PTFE) emulsion (60 wt%), 0.1 g PPG-co-PETA/LAGP composite powder was dispersed. With grinding and solvent volatilization, a soft slurry was obtained and then firmly pressed into a film with the thickness of ~50 μm. The film was cut into a circular shape with a diameter of 16 mm. The PPG-co-PETA/LAGP membranes were dried to remove the residual solvent in a vacuum oven at 60 °C for 24 h to be used to characterize electrochemical properties.

### 2.4. Material Characterizations

The crystal structure of LAGP and PPG-co-PETA/LAGP composite were measured by X-ray diffraction (XRD, Bruker D8 ADVANCE, Germany) from 5° to 60° with a scanning rate of 6° min^−1^ at room temperature. The morphologies, elements’ composition and elements’ distribution were observed by scanning electron microscopy (SEM, Hitachi S-4800, Japan) equipped with energy dispersive X-ray spectroscopy (EDS). Thermogravimetric analysis (TGA, TGA-50) was carried out under an oxygen atmosphere from 30 to 600 °C with the heating rate of 5 °C min^−1^ to determine the amount of LAGP in the composite. The nitrogen adsorption/desorption measurement was carried out to characterize the specific surface area (S_BET_) and the pore size distribution, which were calculated by Brunauer-Emmett-Teller (BET) theory and density functional theory (DFT) method respectively, on a micromeritics analyzer (ASAP 2020M, Micromeritics, Norcross, GA, USA). Atomic force microscopy (AFM, Bruker Multimode 8, Germany) was tested by peak force quantitative mechanical mapping to characterize the mechanical properties of electrolyte membranes. The electrolyte uptake of the PPG-co-PETA/LAGP QSSCE was calculated from the following Equation (1), where *w*_0_ is the initial weight of the QSSCE, and *w_i_* is the final weight of the QSSCE after absorbing electrolytes for 6 h:

(1) $$Electrolyte\;uptake\;\left(\%\right)=\frac{w_{i}-w_{0}}{w_{0}}\times 100\%\;\;\;$$

### 2.5. Li-S Battery Assembly

The cathode slurry composed of super-P/S composite (80 wt%, the synthesis and the sulfur content are shown in Supplementary Figure S1), acetylene black (10 wt%) and polyvinylidene fluoride (PVDF, 10 wt%) in N-methyl pyrrolidone (NMP) solvent was coated on the carbon-coated aluminum foil and dried in a vacuum oven at 65 °C for 24 h. The working cathode was obtained by cutting foil into a circular shape with a diameter of 12 mm. The CR2032 coin cell was assembled with cathode, PPG-co-PETA/LAGP QSSCE, lithium anode and an appropriate amount of liquid electrolyte in an argon-filled glove box (H_2_O < 0.1 ppm, O_2_ < 0.1 ppm). The liquid electrolyte was composed of 1 M lithium perchlorate (LiClO_4_) and 0.1 M lithium nitrate (LiNO_3_) in 1,3-dioxolane (DOL) and dimethoxyethane (DME) (*v*/*v* = 1/1).

### 2.6. Electrochemical Measurements

The Li-ion conductivity (*σ*) of PPG-co-PETA/LAGP QSSCE was measured by the electrochemical impedance spectroscopy (EIS) method in an electrochemical workstation (CHI760E, CH Instrument, Shanghai, China) with a voltage amplitude of 5 mV at the frequency range of 0.1 Hz to 1 MHz. The value was calculated by the following Equation (2) [37], where *R_b_* and *L* are the bulk resistance and the thickness of the QSSCE respectively, and *A* is the contact area between the QSSCE and the electrode:

(2) $$\sigma =\frac{L}{R_{b}\times A}\;\;\;$$

The Li-ion transference number (*t^+^*) of PPG-co-PETA/LAGP QSSCE was characterized by the same electrochemical workstation and calculated by the following Equation (3) [38], where *I*_0_ and *I_S_* are the initial and steady-state currents respectively, ΔV is the applied potential of 10 mV and *R*_0_ and *R_S_* are initial and steady-state interfacial resistance values, which can be obtained from variation of current with time and the EIS curves before and after polarization for 3600 s:

(3) $$t^{+}=\frac{I_{S}\left(\Delta V-I_{0}R_{0}\right)}{I_{0}\left(\Delta V-I_{S}R_{S}\right)}\;$$

The electrochemical stability window was tested by linear sweep voltammetry (LSV) measurement under a scan rate of 5 mV s^−1^, from 0.0 to 6.0 V (vs. Li/Li^+^), and the cyclic voltammetry (CV) was carried out at a scan rate of 0.1 mV s^−1^ between 1.6 and 2.8 V to characterize the redox reaction and reversibility in the same chemical workstation. The galvanostatic charge/discharge cycling performances of the Li-S battery were performed on a battery testing system (CT2001A, LAND, Wuhan, China) under the voltage range of 1.6~2.8 V.

## 3. Results and Discussion

### 3.1. Morphology and Structure of LAGP

**Figure 1:**
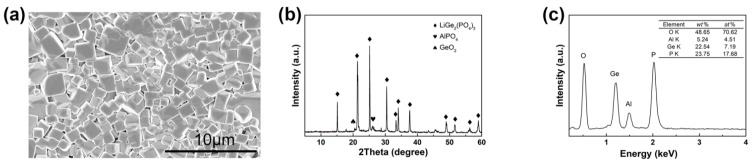
(**a**) SEM image, (**b**) XRD pattern and (**c**) EDS spectrum with the table showing the ratio of elements of self-made LAGP.

The morphology and structure of self-made LAGP were characterized by scanning electron microscopy (SEM), X-ray diffraction (XRD) and energy dispersive spectrometer (EDS). As shown in Figure 1a, LAGP was a regular cubic crystal with the size between 1.0 and 2.0 μm. The XRD pattern of the LAGP (Figure 1b) showed very small amounts of AlO_4_ phases found at 26° and 27°, and GeO_2_ phase found at 21°. All the main peaks of LAGP powder matched well with the pattern of LiGeO_2_(PO_4_)_3_ with NASICON-type structure [39]. The ratio of elements of O, Al, Ge and P identified by the analysis of the EDS (Figure 1c) was found to be in accordance with the stoichiometric ratio of Li_1.5_Al_0.5_Ge_1.5_(PO_4_)_3_.

### 3.2. Morphology and Structure of the PPG-co-PETA/LAGP Composite

After the extraction with acetone in a Soxhlet extractor, the unreacted or un-crosslinked byproducts were removed, which changed the contents of LAGP in PPG-co-PETA/LAGP composite, compared with the initial value. The actual contents of LAGP were measured by thermogravimetric analysis (TGA) at an O_2_ atmosphere, as shown in Supplementary Figure S2. The LAGP almost had no mass loss at 600 °C, while a small loss of 1.11% belonged to the adsorbate (such as moisture) on the surface of LAGP. For all PPG-co-PETA/LAGP composites, the polymer component was completely removed by heating under O_2_ atmosphere, leaving the LAGP component. Compared with the result of PPG-co-PETA and LAGP, the actual contents of LAGP in PPG-co-PETA/LAGP composite were calculated, as shown in Supplementary Table S2. The crystallizations of PPG-co-PETA, LAGP and PPG-co-PETA/LAGP composites were measured by XRD characterization, as shown in Supplementary Figure S3. The characteristic peak of PPG-co-PETA was broad, indicating that PPG-co-PETA was an amorphous structure. After adding LAGP powder, the crystal characteristic peaks of LAGP were shown significantly, and the position of these peaks remained unchanged in PPG-co-PETA/LAGP composite. With the increase of LAGP content, the intensity of peaks was also increasing. The crystal structure of LAGP in composite had never been changed during the in situ UV curing synthesis.

**Figure 2:**
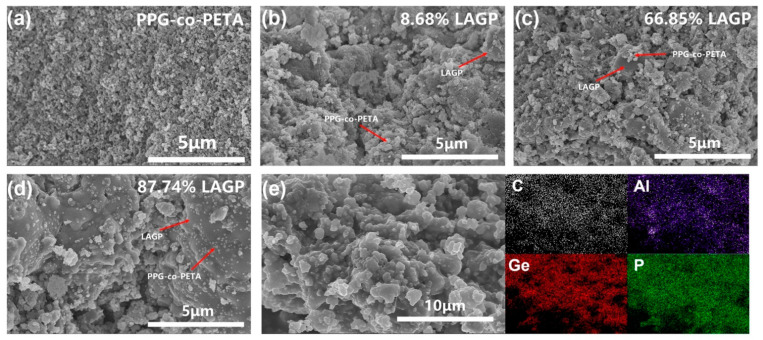
SEM images of (**a**) PPG-co-PETA and (**b**–**d**) composite PPG-co-PETA/LAGP with different content of LAGP. (**e**) Elemental mapping images of composite PPG-co-PETA/LAGP.

The morphological characteristics of the PPG-co-PETA/LAGP composites with different content of LAGP were characterized by SEM, as shown in Figure 2a–d. Figure 2a shows the morphology of PPG-co-PETA without the addition of LAGP powder, whose particle size was about 50 nm. As Figure 2b–d show, PPG-co-PETA was formed on the surface LAGP during the in situ UV curing. In Figure 2b, when the content of LAGP was particularly low, it was difficult to observe the morphology of LAGP in composite, because LAGP particles were wrapped by PPG-co-PETA. With the increase of LAGP content, the particle texture of LAGP gradually became obvious. However, when the content of LAGP reached up to 87.74%, the particle of LAGP was exposed and aggregated, which led to direct contact between LAGP and the electrodes, and this was not conducive to the reduction of interfacial impedance with electrodes. To further assess the composition distribution in the PPG-co-PETA/LAGP composite, element mappings of composites were measured by EDS, as shown in Figure 2e. The element of C belonged to PPG-co-PETA, and the elements of Al, Ge and P belonged to LAGP. All the elements were distributed evenly in the composite, indicating that the PPG-co-PETA and LAGP were distributed evenly without aggregation.

### 3.3. The Liquid Electrolyte Uptake and Electrochemical Performance of the PPG-co-PETA/LAGP QSSCE

**Figure 3:**
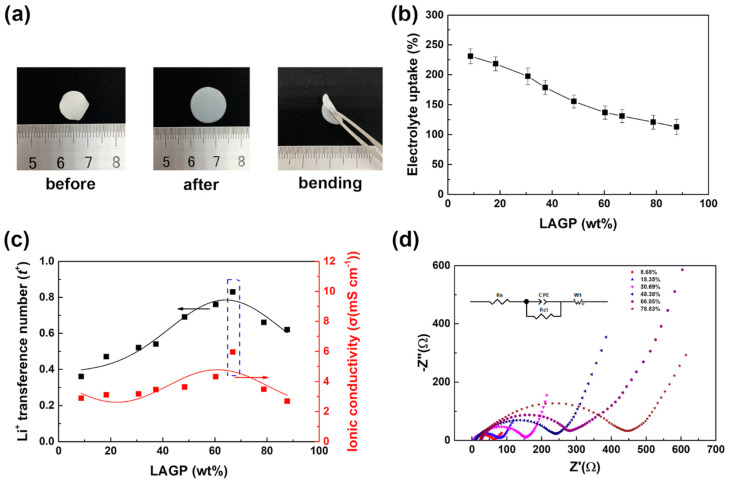
(**a**) The digital photographs of the QSSCE before and after absorbing liquid electrolyte showing the flexibility, (**b**) the liquid electrolyte uptake, (**c**) Li-ion conductivity and Li-ion transference number and (**d**) EIS curves of the QSSCE with different content of LAGP, inserted with the corresponding equivalent electrical circuit.

All the PPG-co-PETA/LAGP composites were prepared into QSSCE membranes to characterize their ability of liquid electrolyte uptake and electrochemical properties. The liquid component can not only provide a proper development of kinetically faster electrochemical reactions, but also wet LAGP to reduce the interfacial impedance with electrodes. As shown in Figure 3a, the diameter of QSSCE membrane swelled from 1.0 to 1.5 cm after absorbing liquid electrolyte. Besides, the QSSCE membrane could be bent, indicating that it could be used for flexible devices. The liquid electrolyte uptake of the PPG-co-PETA/LAGP composite QSSE is shown in Figure 3b. The nanopore structure of PPG-co-PETA provided the ability of absorbing liquid electrolyte for QSSCE, while LAGP was not available for liquid electrolyte absorption, so that the reduction of the content of PPG-co-PETA resulted in the decrease of the liquid electrolyte uptake for QSSCE. Although the liquid electrolyte uptake of the QSSCE decreased with the increase of LAGP, it was still higher than 100%, indicating a good wettability and adsorption for the liquid electrolyte.

In terms of electrochemical properties, Li-ion transference number (*t^+^*) and Li-ion conductivity (*σ*) are the critical parameters of QSSCE in the Li-S battery. The *t^+^* indicates the contribution of the different ions toward the total current carried by the electrolyte, and the *σ* indicates the transmission dynamics of Li-ion. The increase of *t^+^* and *σ* is beneficial to improve the electrochemical performances of the Li-S battery. The *t^+^* and *σ* of QSSCE with different content of LAGP were characterized by the electrochemical impedance spectroscopy (EIS) method in a symmetric coin cell (Li/electrolyte/Li), as shown in Figure 3c, and Supplementary Figure S4 and Table S3. It could be observed that when the content of LAGP increased from 8.68% to 66.85%, the values of *t^+^* and *σ* of QSSCE increased from 0.36 to 0.83 and from 3.02 × 10^−3^ to 5.95 × 10^−3^ S cm^−1^, respectively. The addition of LAGP facilitated the transmission of Li-ion, due to the high *t^+^* and *σ* of LAGP. However, with the content of LAGP increasing further, both the values of *t^+^* and *σ* decreased. As indicated above, when the content of LAGP was greater than 66.85%, most LAGP particles were exposed to the surface in composite (Figure 2d), which enhanced the interfacial resistance between QSSCE and electrodes. To characterize the interfacial impedance, the Nyquist plots of all electrolytes were tested via EIS measurement, as shown in Figure 3d and Supplementary Figure S5, inserted with the corresponding equivalent circuit. The corresponding plots of all electrolytes could be well-fitted into the equivalent electrical circuit, where Rs stands for the internal resistance of the electrolyte, Rct represents the resistance of charge transfer, W1 is the Warburg resistance and the CPE corresponds to the constant phase element. Normally, the EIS curve is divided into two parts. One part is a semicircle in the high-frequency region, due to the bulk impedance and the interface impedance, and the other part is an oblique line in the low-frequency region, due to the diffusion process of Li^+^ [40,41]. It was noteworthy that the bulk impedances of QSSCE with different content of LAGP were quite similar, while the interface impedance increased significantly, with the increase of the content of LAGP, from 44.9 Ω (QSSCE without LAGP) to 1629.4 Ω (LAGP electrolyte), indicating that the addition of LAGP increased the interface impedance of QSSCE with electrodes due to exposure of LAGP on the surface. In addition, the composite with higher LAGP showed lower liquid electrolyte uptake (Figure 3b), which might also be the reason for the decreased lithium-ion transference number and ion conductivity. Therefore, when the content of LAGP reached 66.85%, the highest *t^+^* and *σ* values of 0.83 and 5.95 × 10^−3^ S cm^−1^ respectively, for QSSCE were obtained.

As a new type of electrolytes, in addition to the excellent Li-ion conductivity, the PPG-co-PETA/LAGP QSSCE has to possess a high electrochemical stability window to meet the requirements of its application in Li-S batteries. Supplementary Figure S6 showed that the electrochemical stability of all electrolytes measured by linear sweep voltammetry (LSV) was over 4.0 V, meaning that all electrolytes could meet the requirements for the Li-S battery. The electrochemical stability of the PPG-co-PETA was about 4.5 V (vs. Li/Li^+^), and with the increase of the content of LAGP, the electrochemical stability of the QSSCE gradually increased to 6.0 V (vs. Li/Li^+^), indicating that the addition of LAGP powder could effectively improve the electrochemical stability of the QSSCE.

**Figure 4:**
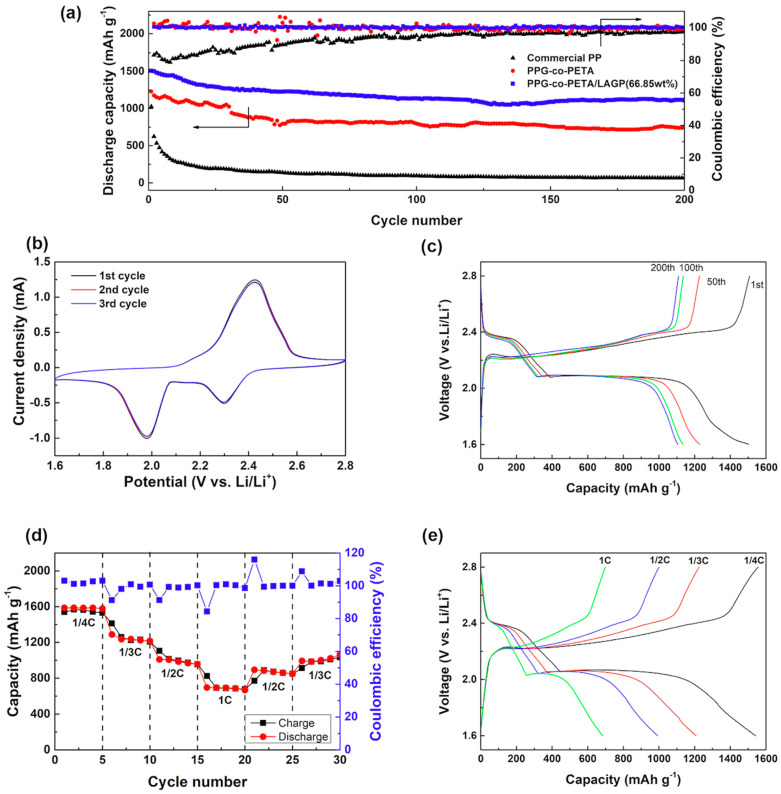
(**a**) The cycling performances of the Li-S battery using QSSCE compared with using PPG-co-PETA electrolyte and PP separator at the current of 0.25 C. (**b**) The CV curves of the QSSCE, collected in the potential from 1.6 to 2.8 V (vs. Li/Li^+^) at a scan rate of 0.1 mV/s. (**c**) The galvanostatic charge/discharge curves of the QSSCE at the current of 0.25 C at different cycles. (**d**) The rate performances of the QSSCE. (**e**) The charge/discharge curves of the QSSCE under the currents of 1/4, 1/3, 1/2 and 1 C, respectively.

Through the above tests of electrochemical performance, the QSSCE with 66.85% LAGP was selected to assemble the Li-S battery due to its highest values of *t^+^* and *σ*, the favorable electrochemical stability window as well as sufficient liquid electrolyte uptake. The cycling performances of a Li-S battery using the PPG-co-PETA/LAGP QSSCE between the voltages of 1.6 and 2.8 V are shown in Figure 4. As control samples, PPG-co-PETA and commercial PP separator (Celgard 2400) were also assembled into batteries and measured under the same conditions. However, the LAGP electrolyte membrane could not be assembled since it was easy to destroy due to its fragileness. According to Figure 4a, it could be seen that the discharge capacities of PPG-co-PETA/LAGP, of PPG-co-PETA and of PP were dropped from 1508.1, 1229.4 and 1026.0 mAh g^−1^, to 1109.5, 746.7 and 67.7 mAh g^−1^ after 200 cycles, respectively. Obviously, the cycling performances of the battery using QSSCE and PPG-co-PETA were much superior to that using the PP separator, with a coulombic efficiency ≥ 98%. Moreover, the discharge capacity of the battery using QSSCE was also better than using PPG-co-PETA, indicating that the addition of LAGP could improve the cycling performance of the Li-S battery. To further investigate the Li-S battery performance, the cycle-voltammogram of the QSSCE was collected in the potential from 1.6 to 2.8 V (vs. Li/Li^+^) at a scan rate of 0.1 mV s^−1^, as shown in Figure 4b. There were two reduction peaks and one oxidation peak, obviously, which were attributed to the typical multi-step reaction mechanism of the Li-S battery. The higher reduction peak at 2.30 V was attributed to the reduction of the ring element sulfur (S_8_) to the soluble long-chain polysulfides Li_2_S_x_ (4 ≤ x ≤ 8), and the lower one at 1.98 V accounted for the reduction of soluble polysulfides to the insoluble short-chain polysulfides Li_2_S_2_ and Li_2_S. Correspondingly, the oxidation peak at 2.42 V corresponded to the converse oxidation reactions of the short-chain polysulfides Li_2_S_2_ and Li_2_S to the long-chain polysulfides Li_2_S_x_ (4 ≤ x ≤ 8), and finally to the sulfur (S_8_). During the cycling, the curves were over-lapped without remarkable potential shift, indicating that the Li-S battery using QSSCE possessed a good reversibility and promising cycling stability. Similarly, the galvanostatic charge/discharge curves at 0.25 C, shown in Figure 4c, also validated this conclusion. The Li-S battery presented a charge platform at ~2.20 V, and two discharge platforms at ~2.35 and ~2.10 V, representing the typical redox reaction of sulfide oxidation and two-step sulfur reduction, which were consistent with the oxidation peak and two reduction peaks in the CV curves. There were slight changes in the charge and discharge profiles after 50, 100 and even 200 cycles, and the charge and discharge capacity of the battery were equivalent, indicating that the QSSCE could more effectively prevent the polysulfides shuttle effect. In addition, the Li-S battery using the QSSCE exhibited an excellent charge/discharge performance at a different current rate, ranging from 0.25 to 1 C, as shown in Figure 4d. When the current rate increased from 0.25 to 1 C, the Li-S battery delivered the reduced discharge capacity of 1233.7 to 546.37 mAh g^−1^. Moreover, as the current rate went back to 1/3 C, the discharge capacity could be recovered to about 850.7 mAh g^−1^, showing the great reversibility. Figure 4e shows the galvanostatic charge/discharge curves of the Li-S battery at various current rates ranging from 0.25 to 1 C. The shapes of all the curves were similar, indicating that the same electrochemical reactions happened during the charge/discharge process at different current rates.

### 3.4. Exploring the Mechanism of the PPG-co-PETA/LAGP QSSCE

**Figure 5:**
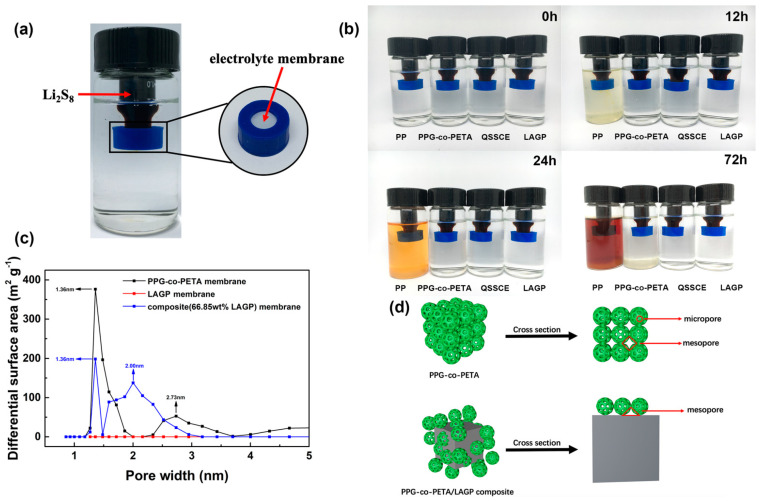
(**a**) The self-made device for characterizing the ability of polysulfides’ shuttle effect. (**b**) The polysulfides’ diffusion test of PP, PPG-co-PETA, QSSCE and LAGP at different times. (**c**) The pore size distribution curves of PPG-co-PETA, QSSCE and LAGP. (**d**) The schematic drawing of the micropore and mesopore structure of the PPG-co-PETA and PPG-co-PETA/LAGP composites.

To explore the mechanism of the PPG-co-PETA/LAGP QSSCE on the performance improvement of the Li-S battery, the shuttle effect of the Li-S battery and mechanical property of the QSSCE were characterized. In order to manifest the ability of the QSSCE to inhibit the shuttle effect of polysulfides, the visualization experiment was carried out in a self-made device, as shown in Figure 5a. The large sample vial was filled with blank liquid electrolyte, 1 M LiClO_4_ in DME and DOL (*v*/*v* = 1/1), and the small sample vial was filled with 50 mM Li_2_S_8_ solution, synthesized via the reacting element sulfur (S) and lithium sulfide (Li_2_S) in the desired ratio under stirring inside an argon-filled glove box at room temperature. The electrolyte membrane was located on the lid of the small sample vial to block the diffusion of the polysulfide (Li_2_S_8_). To form a control group, we compared the QSSCE with the commercial PP separator, PPG-co-PETA electrolyte and LAGP electrolyte respectively, as shown in Figure 5b. At the initial stage of the experiment, the solutions in all large bottles were transparent and colorless. After 12 h, a slight penetration occurred in the PP separator, indicating that the PP separator was unable to suppress the polysulfide shuttle, while there were no polysulfide penetrations in the other three vials. After 24 h, the yellow color of liquid electrolyte in the PP separator vial became darker and darker, showing significant penetration, and the other three were still colorless. With the increase of time, the contrast between the PP separator and the other three became more obvious. The solution outside the PP separator became more turbid. However, there was a little polysulfide penetration in the PPG-co-PETA electrolyte, while the QSSCE and LAGP electrolytes remained transparent and colorless, indicating that the introduction of LAGP into PPG-co-PETA was beneficial to inhibit the polysulfide shuttle. It has been reported that the particle size of Li_2_S_8_ polysulfide is ~2.2 nm during the discharge/charge process [42]. The PP separator was completely unable to suppress the polysulfide shuttle due to the fact that its pore size (20~50 nm) was much larger than the particle size of polysulfide. To measure the pore size of PPG-co-PETA, LAGP and composite electrolytes, the pore size distribution curves of them were measured by N_2_ adsorption measurements and analyzed by the density functional theory (DFT) method, as shown in Figure 5c. It could be observed that both PPG-co-PETA and QSSCE possessed micropore structure with the pore size of ~1.36 nm, while the pore sizes of mesopore for PPG-co-PETA and QSSCE were ~2.73 and ~2.00 nm, respectively. The pore size of mesopore for the PPG-co-PETA electrolyte was larger than the particle size of polysulfide, which accounted for the fact that the polysulfide penetration occurred in the PPG-co-PETA bottle but not in QSSCE after a long time. The mesopore was formed by stacked PPG-co-PETA particles. It could be inferred that after adding LAGP powder, the aggregation of PPG-co-PETA particles was reduced by loading on the surface of LAGP particles evenly, as shown in the schematic drawing of Figure 5d. In addition, LAGP particles also occupied part of the space of the mesopore, so that the mesopore size of QSSCE decreased, inhibiting the shuttle effect more effectively. For the LAGP electrolyte, it can block the polysulfide shuttle completely without porous structure. Therefore, the addition of LAGP ceramic powder not only kept the porous structure of PPG-co-PETA intact during the in situ UV curing process, but also enhanced the ability to suppress polysulfides’ shuttle of QSSCE, which could be well applied for Li-S batteries.

**Figure 6:**
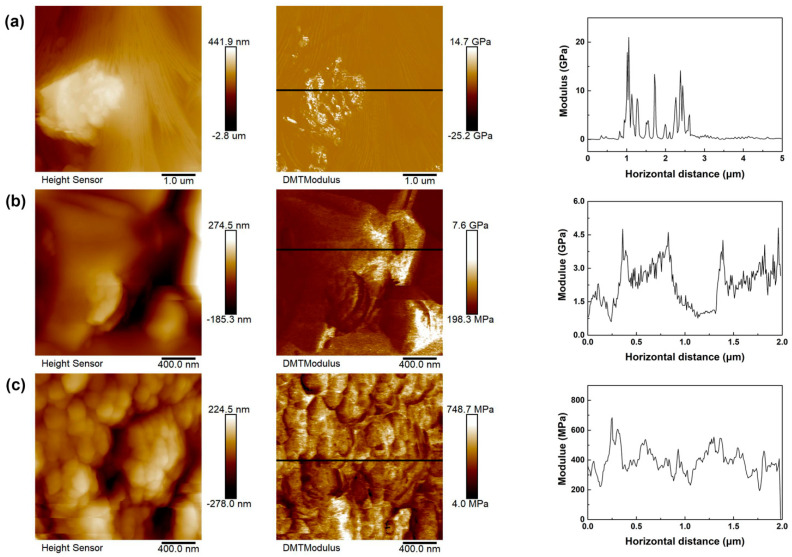
The height images, DMT modulus images with corresponding representative cross-sections and the surface DMT modulus values of (**a**) LAGP, (**b**) QSSCE and (**c**) PPG-co-PETA.

Besides functioning as an electrolyte, the PPG-co-PETA/LAGP QSSCE acting as a separator between electrodes at the same time also needs good mechanical properties to inhibit lithium dendrites to prevent short-circuiting of Li-S batteries. To further study the mechanical properties of electrolytes, the morphology and the surface DMT modulus values of LAGP electrolyte, QSSCE and PPG-co-PETA electrolyte were measured by atomic force microscopy (AFM) under the Peak Force Tapping mode, as shown in Figure 6, including the cross-section representing the typical ranges of a DMT modulus value. It has been reported that the modulus of lithium was 3.4 GPa, so that the formation of lithium dendrites can be effectively inhibited by using an electrolyte whose modulus is higher than or even twice that of lithium [43,44,45]. It could be known from the DMT modulus image of Figure 6a that the modulus of LAGP was up to ~21 GPa, much higher than the modulus of lithium, indicating that the LAGP electrolyte can completely suppress the formation of lithium dendrites. As Figure 6b,c show, the modulus of QSSCE was up to ~4.8 GPa, also larger than that of lithium, while the modulus of the PPG-co-PETA electrolyte was only ~0.7 GPa (Figure 6c), which failed to meet the requirement of suppressing the lithium dendrites. These results suggested that the addition of LAGP could enhance the mechanical properties of QSSCE, thereby inhibiting the formation of lithium dendrites more effectively.

**Figure 7:**
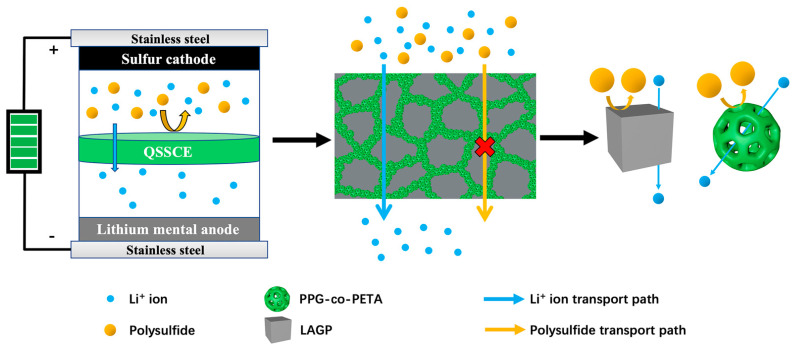
The mechanistic schematic drawing of the QSSCE for suppressing polysulfides’ shuttle effect in the Li-S battery.

Based on the above investigations, the possible mechanism of QSSCE in accelerating Li-ion transportation and performance improvement of the Li-S battery is revealed by the schematic drawing of Figure 7. The prepared PPG-co-PETA/LAGP composite was composed of polymer PPG-co-PETA and inorganic solid LAGP, and PPG-co-PETA was formed on the surface of LAGP (based on observations in Figure 2). The QSSCE showed excellent swelling ability due to the ability of PPG-co-PETA to absorb liquid electrolytes, which could reduce the interfacial impedance between QSSCE and electrodes (according to the results shown in Figure 3a,b,d). The excellent Li-ion conductivity of QSSCE was attributed to not only the inherent good Li-ion conductivity of LAGP but also the amorphous structure of PPG-co-PETA, which was available for the promotion of Li-ion transportation (according to results of Figure 3c). For suppressing polysulfides’ shuttle effect, the synergism of the micropore structure of PPG-co-PETA and the physical barrier of LAGP played an important role (according to the visualization experiment and DFT result shown in Figure 5). As shown by these results, blending polymer PPG-co-PETA with inorganic solid LAGP to obtain QSSCE not only possessed a remarkable Li-ion conductivity, but also effectively inhibited polysulfides’ diffusion, which significantly enhanced the cycling performance of the Li-S battery (according to the results of Figure 4).

## 4. Conclusions

In this work, a novel blended composite of poly(propylene glycol)-co-pentaerythritol triacrylate/Li_1.5_Al_0.5_Ge_1.5_(PO_4_)_3_ (PPG-co-PETA/LAGP) for quasi-solid-state composite electrolyte (QSSCE) was prepared by the in situ UV curing method and used in a Li-S battery. The morphology and structure of PPG-co-PETA/LAGP composite was characterized by XRD and SEM. The results showed that cross-linked polymer PPG-co-PETA was formed on the surface of LAGP particles, and the crystal structure of LAGP was not altered during the process. The relationship between the electrochemical properties of PPG-co-PETA/LAGP QSSCE and the content of LAGP was investigated. The flexible PPG-co-PETA/LAGP QSSCE with a LAGP content of 66.85% possessed the highest Li-ion conductivity (*σ*), of 5.95 × 10^−3^ S cm^−1^, and Li-ion transference number (*t^+^*) of 0.83, as well as the large electrochemical stability of ~5.0 V (vs. Li/Li^+^). With the addition of LAGP, PPG-co-PETA/LAGP QSSCE could inhibit the shuttle effect of polysulfides and effectively suppress the formation of lithium dendrites. The Li-S battery using the PPG-co-PETA/LAGP QSSCE with a LAGP content of 66.85% exhibited more stable cycling performance (capacity retention rate of 73.6% after 200 cycles at 0.25 C, with the low-capacity degradation of 0.13% per cycle). It is believed that PPG-co-PETA/LAGP QSSCE has the potential to promote the development of the Li-S battery, especially for flexible devices.

## Data Availability

The data presented in this study are available on request from the corresponding author.

## Supplementary Materials

The following are available online at https://www.mdpi.com/article/10.3390/ma14081979/s1, Figure S1: The thermogravimetric (TG) curve of super-P/sulfur composite. Figure S2: The thermogravimetric (TG) curves of (a) the PPG-co-PETA and LAGP under an O_2_ atmosphere, and (b) the PPG-co-PETA/LAGP composite with different LAGP content under an O_2_ atmosphere. Figure S3: The XRD patterns of PPG-co-PETA, LAGP and PPG-co-PETA/LAGP composites. Figure S4: Variation of current with time during polarization of QSSCE with different content of LAGP at the applied potential of 10 mV, inserted with the impedance spectra before and after polarization for 3600 s, and the corresponding equivalent circuit, where Rs stands for the internal resistance of the electrolyte, Rct represents the resistance of charge transfer and the CPE is the constant phase element. Figure S5: EIS curves of the QSSCE with different content of LAGP, inserted with the corresponding equivalent electrical circuit. Figure S6: The electrochemical stability curves of all electrolytes measured by the linear sweep voltammetry (LSV) method. Table S1: Components of synthesized PPG-co-PETA/LAGP composite. Table S2: The different actual content of LAGP in PPG-co-PETA/LAGP composite. Table S3: The Li-ion transference number (*t^+^*) of QSSCE with different content of LAGP.

## References

[1] Armand M., Tarascon J.M. (2008). Building Better Batteries. Nature.

[2] Zachos J., Pagani M., Sloan L., Thomas E., Billups K. (2001). Trends, Rhythms, and Aberrations in Global Climate 65 Ma to Present. Science.

[3] Larcher D., Tarascon J.M. (2015). Towards Greener and More Sustainable Batteries for Electrical Energy Storage. Nat. Chem..

[4] Evers S., Nazar L.F. (2012). New Approaches for High Energy Density Lithium-Sulfur Battery Cathodes. Acc. Chem. Res..

[5] Lin Z., Liang C. (2015). Lithium-Sulfur Batteries: From Liquid to Solid Cells. J. Mater. Chem. A.

[6] Lin C., Chen W., Song Y., Wang C., Tsai L., Wu N. (2014). Understanding Dynamics of Polysulfide Dissolution and Re-Deposition in Working Lithium-Sulfur Battery by In-Operando Transmission X-Ray Microscopy. J. Power Sources.

[7] Wild M., O’Neill L., Zhang T., Purkayastha R., Minton G., Marinescu M., Offer G.J. (2015). Lithium Sulfur Batteries, A Mechanistic Review. Energy Environ. Sci..

[8] Lei T., Chen W., Lv W., Huang J., Zhu J., Chu J., Yan C., Wu C., Yan Y., He W. (2018). Inhibiting Polysulfide Shuttling with a Graphene Composite Separator for Highly Robust Lithium-Sulfur Batteries. Joule.

[9] Hao Y., Wang S., Xu F., Liu Y., Feng N., He P., Zhou H. (2017). A Design of Solid-State Li-S Cell with Evaporated Lithium Anode to Eliminate Shuttle Effects. ACS Appl. Mater. Interfaces.

[10] Gu S., Qian R., Jin J., Wang Q., Guo J., Zhang S., Zhuo S., Wen Z. (2016). Suppressing The Dissolution of Polysulfides with Cosolvent Fluorinated Diether towards High-performance Lithium Sulfur Batteries. Phys. Chem. Chem. Phys..

[11] Sun C., Liu J., Gong Y., Wilkinson D.P., Zhang J. (2017). Recent Advances in All-Solid-State Rechargeable Lithium Batteries. Nano Energy.

[12] Ding B., Wang J., Fan Z., Chen S., Lin Q., Lu X., Dou H., Nanjundan A.K., Gleb Y., Zhang X. (2020). Solid-State Lithium-Sulfur Batteries: Advances, Challenges and Perspectives. Mater. Today.

[13] Guo J., Du X., Zhang X., Zhang F., Liu J. (2017). Facile Formation of a Solid Electrolyte Interface as a Smart Blocking Layer for High-Stability Sulfur Cathode. Adv. Mater..

[14] Wang P., Bao J., Lv K., Zhang N., Chang Z., He P., Zhou H. (2019). Rational Design of a Gel-Polymer-Inorganic Separator with Uniform Lithium-Ion Deposition for Highly Stable Lithium-Sulfur Batteries. ACS Appl. Mater. Interfaces.

[15] Xu R., Xiao Y., Zhang R., Cheng X., Zhao C., Zhang X., Yan C., Zhang Q., Huang J. (2019). Dual-Phase Single-Ion Pathway Interfaces for Robust Lithium Metal in Working Batteries. Adv. Mater..

[16] Breuer S., Prutsch D., Ma Q., Epp V., Preishuber-Pflugl F., Tietz F., Wilkening M. (2015). Separating Bulk from Grain Boundary Li Ion Conductivity in the Sol-Gel Prepared Solid Electrolyte Li_1.5_Al_0.5_Ti_1.5_(PO_4_)_3_. J. Mater. Chem. A.

[17] Thangadurai V., Weppner W. (2006). Recent Progress in Solid Oxide and Lithium Ion Conducting Electrolytes Research. Inoics.

[18] O’Callaghan M.P., Powell A.S., Titman J.J., Chen G.Z., Cussen E.J. (2008). Switching on Fast Lithium Ion Conductivity in Garnets: The Structure and Transport Properties of Li_3+x_Nd_3_Te_2−x_Sb_x_O12. Chem. Mater..

[19] Stramare S., Thangadurai V., Weppener W. (2003). Lithium Lanthanum Titanates: A Review. Chem. Mater..

[20] Ma Z., Xue H., Guo S. (2018). Recent Achievements on Sulfide-Type Solid Electrolytes: Crystal Structures and Electrochemical Performance. J. Mater. Sci..

[21] Quartarone E., Mustarelli P. (2011). Electrolytes for Solid-State Lithium Rechargeable Batteries: Recent Advances and Perspectives. Chem. Soc. Rev..

[22] Agrawal R.C., Pandey G.P. (2008). Solid Polymer Electrolytes: Materials Designing and All-Solid-State Battery Applications: An Overview. J. Phys. D Appl. Phys..

[23] Chen R., Li Q., Yu X., Chen L., Li H. (2020). Approaching Practically Accessible Solid-State Batteries: Stability Issues Related to Solid Electrolytes and Interfaces. Chem. Rev..

[24] Wang S., Xu H., Li W., Dolocan A., Manthiram A. (2018). Interfacial Chemistry in Solid-State Batteries: Formation of Interphase and Its Consequences. J. Am. Chem. Soc..

[25] Li Y., Xu B., Xu H., Duan H., Lü X., Xin S., Zhou W., Xue L., Fu G., Manthiram A. (2017). Hybrid Polymer/Garnet Electrolyte with a Small Interfacial Resistance for Lithium-Ion Batteries. Angew. Chem. Int. Ed..

[26] Judez X., Martinez-Ibanez M., Santiago A., Armand M., Zhang H., Li C. (2019). Quasi-Solid-State Electrolytes for Lithium Sulfur Batteries: Advances and Perspectives. J. Power Sources.

[27] Qian J., Jin B., Li Y., Zhan X., Hou Y., Zhang Q. (2021). Research Progress on Gel Polymer Electrolytes for Lithium-Sulfur Batteries. J. Energy Chem..

[28] Wang Q., Jin J., Wu X., Ma G., Yang J., Wen Z. (2014). A Shuttle Effect Free Lithium Sulfur Battery Based on a Hybrid Electrolyte. Phys. Chem. Chem. Phys..

[29] Jin J., Wen Z., Wang Q., Gu S., Huang X., Chen C. (2016). Protected Sulfur Cathode with Mixed Conductive Coating Layer for Lithium Sulfur Battery. JOM.

[30] Wang Q., Guo J., Wu T., Jin J., Yang J., Wen Z. (2017). Improved Performance of Li-S Battery with Hybrid Electrolyte by Interface Modification. Solid State Ion..

[31] Wang Q., Wen Z., Jin J., Guo J., Huang X., Yang J., Chen C. (2016). A Gel-Ceramic Multi-Layer Electrolyte for Long-Life Lithium Sulfur Batteries. Chem. Commun..

[32] Gu S., Huang X., Wang Q., Jin J., Wang Q., Wen Z., Qian R. (2017). A Hybrid Electrolyte for Long-Life Semi-Solid-State Lithium Sulfur Batteries. J. Mater. Chem. A.

[33] Xu H., Wang S., Manthiram A. (2018). Hybrid Lithium-Sulfur Batteries with an Advanced Gel Cathode and Stabilized Lithium-Metal Anode. Adv. Energy Mater..

[34] Diederichsen K.M., McShane E.J., McCloskey B.D. (2017). Promising Routes to a High Li^+^ Transference Number Electrolyte for Lithium Ion Batteries. ACS Energy Lett..

[35] Zheng J., Hu Y. (2018). New Insights into the Compositional Dependence of Li-Ion Transport in Polymer-Ceramic Composite Electrolytes. ACS Appl. Mater. Interfaces.

[36] Li H., Tang W., Huang Y., Ruan W., Zhang M. (2019). Nanopore Separator of Cross-Linked Poly(Propylene Glycol)-co-Pentaerythritol Triacrylate for Effectively Suppressing Polysulfide Shuttling in Li-S Batteries. Polym. Chem..

[37] Zhang J., Zhong H., Zheng C., Xia Y., Liang C., Huang H., Gan Y., Tao X., Zhang W. (2018). All-Solid-State Batteries with Slurry Coated LiNi_0.8_Co_0.1_Mn_0.1_O_2_ Composite Cathode and Li_6_PS_5_Cl Electrolyte: Effect of Binder Content. J. Power Sources.

[38] Liu B., Huang Y., Gao H., Zhao L., Huang Y., Song A., Lin Y., Li X., Wang M. (2018). A Novel Porous Gel Polymer Electrolyte Based on Poly(Acrylonitrile-Polyhedral Oligomeric Silsesquioxane) with High Performances for Lithium-ion Batteries. J. Membr. Sci..

[39] Liu Z., Venkatachalam S., Wullen L. (2015). Structure, Phase Separation and Li Dynamics in Sol-Gel-Derived Li_1+x_Al_x_Ge_2−x_(PO_4_)_3_. Solid State Ion..

[40] Conder J., Villevieille C., Trabesinger S., Novak P., Gubler L., Bouchet R. (2017). Electrochemical Impedance Spectroscopy of a Li-S Battery: Part 1. Influence of the Electrode and Electrolyte Compositions on the Impedance of Symmetric Cells. Electrochim. Acta.

[41] Conder J., Villevieille C., Trabesinger S., Novak P., Gubler L., Bouchet R. (2017). Electrochemical Impedance Spectroscopy of a Li-S Battery: Part 2. Influence of Separator Chemistry on the Lithium Electrode/Electrolyte Interface. Electrochim. Acta.

[42] Vijayakumar M., Govind N., Walter E., Burton S.D., Shukla A., Devaraj A., Liu J., Wang C., Karim A., Thevuthasan S. (2014). Molecular Structure and Stability of Dissolved Lithium Polysulfide Species. Phys. Chem. Chem. Phys..

[43] Zhang X., Wang A., Liu X., Luo J. (2019). Dendrites in Lithium Metal Anodes: Suppression, Regulation, and Elimination. Acc. Chem. Res..

[44] Monroe C., Newman J. (2004). The Effect of Interfacial Deformation on Electrodeposition Kinetics. J. Electrochem. Soc..

[45] Monroe C., Newman J. (2005). The Impact of Elastic Deformation on Deposition Kinetics at Lithium/Polymer Interfaces. J. Electrochem. Soc..

