# Supplementary material for: Flexible Quasi-Solid-State Composite Electrolyte of Poly (Propylene Glycol)-co-Pentaerythritol Triacry-Late/Li1.5Al0.5Ge1.5(PO4)3 for High-Performance Lithium-Sulfur Battery
Source: Materials (Basel). 2021 Apr 15;14(8):1979. doi: 10.3390/ma14081979 (PMC8071246; doi:10.3390/ma14081979)
Supplement: Supplementary file 1 [file materials-14-01979-s001.zip › materials-1178829-supplementary.pdf]

materials

Supplementary Materials

Flexible Quasi-Solid-State Composite Electrolyte of Poly (Propylene Glycol)-co-Pentaerythritol Triacrylate/$Li_{1.5}Al_{0.5}Ge_{1.5}(PO_4)_3$ for High-Performance Lithium-Sulfur Battery

Zekun Deng [1,2], Zhenyang Zheng [1,2], Wenhong Ruan [1,2,*] and Mingqiu Zhang [1,2]

[1] Key Laboratory for Polymeric Composite and Functional Materials of Ministry of Education, School of Chemistry, Sun Yat-sen University, Guangzhou 510275, China; dengzk1996@163.com (Z.D.); zhengzhy8@mail2.sysu.edu.cn (Z.Z.); ceszmq@mail.sysu.edu.cn (M.Z.)
[2] Guangdong Provincial Key Laboratory for High Performance Polymer-based Composites, Guangzhou 510275, China
* Correspondence: cesrwh@mail.sysu.edu.cn; Tel.: +86-020-84114008

Table S1. Components of synthesized PPG-co-PETA/LAGP composite

Component (wt%) / Sample	PO	PETA	DMPA	LAGP(accounting for (PO+PETA+DMPA)mixture)
#1	91.50	7.50	1.00	3.00
#2	91.50	7.50	1.00	5.00
#3	91.50	7.50	1.00	8.00
#4	91.50	7.50	1.00	12.00
#5	91.50	7.50	1.00	15.00
#6	91.50	7.50	1.00	22.00
#7	91.50	7.50	1.00	25.00
#8	91.50	7.50	1.00	30.00
#9	91.50	7.50	1.00	45.00

Preparation of the super-P/S composite

The preparation procedure of the super-P/sulfur composite as follows: Firstly, to introduce massive sulfur element into the super-P (S_{BET} = 2000 m^2 g^{-1}), the procedure is conducted as follows: 2.9 g of

$Na_2S_2O_3 \cdot 5H_2O$ was dissolved in 20mL distilled water containing 0.15 g super-P, then, 15mL 4.20 M HCl was added dropwise into the mixture when stirred, after stirring for 24 h at room temperature, the solution was filtered, washed by distilled water and dried at 60 °C for 24 h, so the resulting super-P/sulfur composite was obtained.

To determine the sulfur content of the composite, the thermography analysis (TGA) was carried out under a nitrogen atmosphere from 30 °C to 600 °C with the heating rate of 5 °C min^{-1}, which showed the sulfur mass percentage was about 63.9 wt%, as shown in Figure S1.

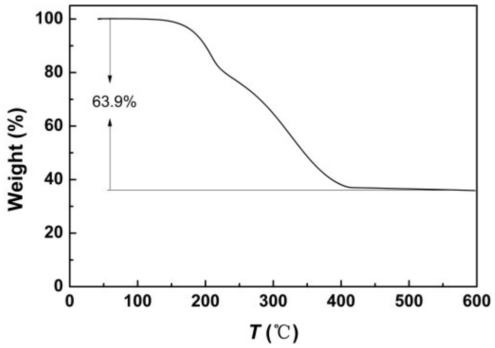

Figure S1. The thermogravimetric (TG) curve of super-P/sulfur composite.

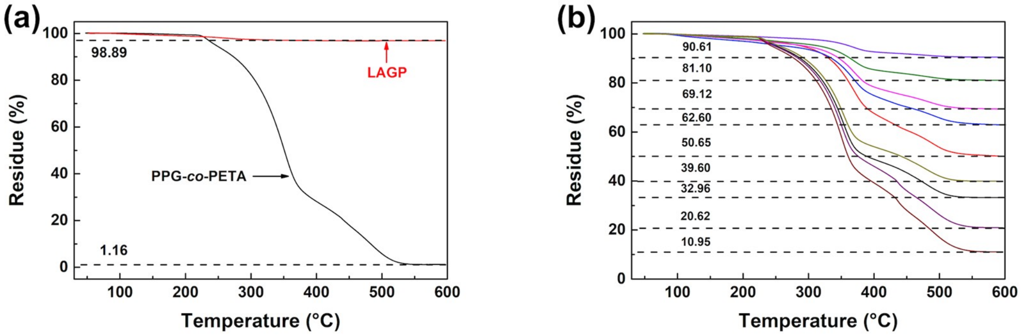

Figure S2. The thermogravimetric (TG) curves of (a) the PPG-co-PETA and LAGP under an O_2 atmosphere, and (b) the PPG-co-PETA/LAGP composite with different LAGP content under an O_2 atmosphere.

Table S2. The different actual content of LAGP in PPG-co-PETA/LAGP composite

Sample	Initial content (%)	Residue (%)	Actual content (%)
LAGP	100.00	98.89	100.00
PPG-co-PETA	100.00	1.16	0.00
#1	3.00	10.95	8.68
#2	5.00	20.62	18.35
#3	8.00	32.96	30.69
#4	12.00	39.62	37.35
#5	15.00	50.65	48.38
#6	22.00	62.60	60.33
#7	25.00	69.12	66.85
#8	30.00	81.10	78.83
#9	45.00	90.01	87.74

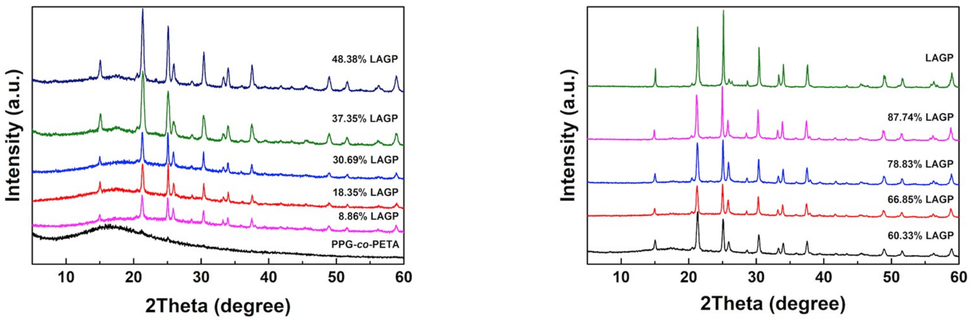

Figure S3. The XRD patterns of PPG-co-PETA, LAGP, and PPG-co-PETA/LAGP composite.

Measurement of the Li-ion transference number

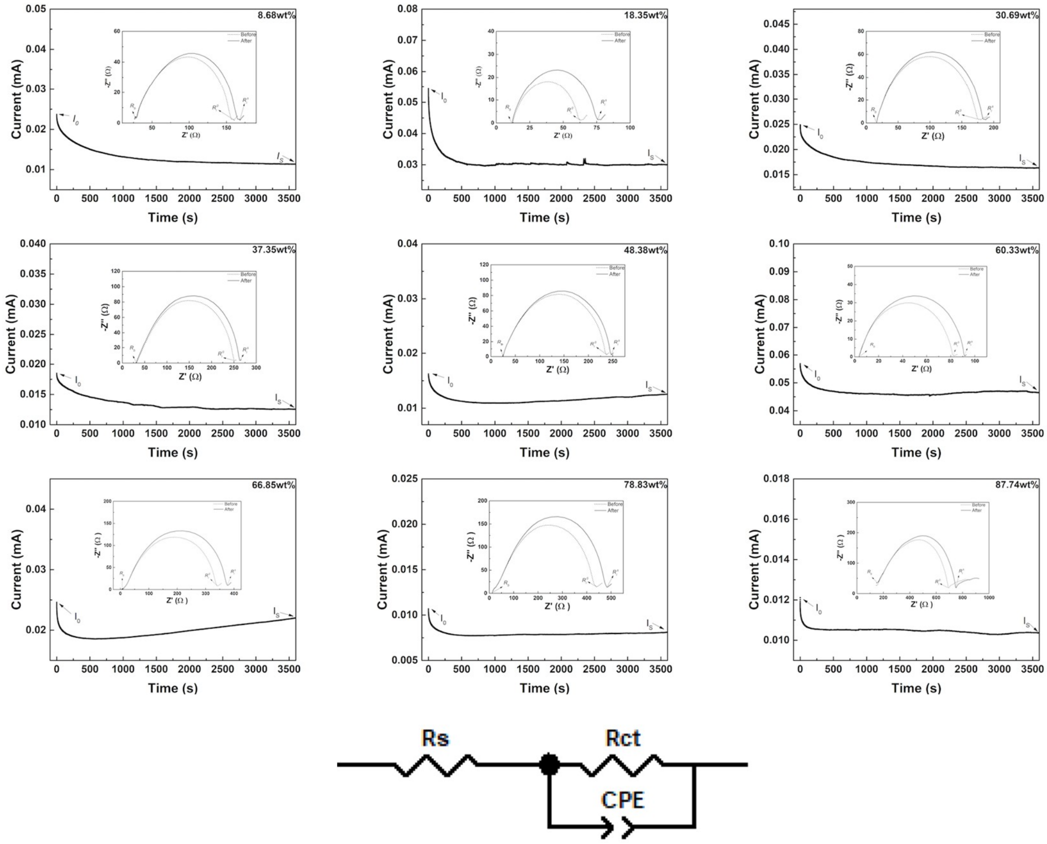

Figure S4. Variation of current with time during polarization of QSSCE with different content of LAGP at the applied potential of 10mV, inserted with the impedance spectra before and after polarization for 3600s, and the corresponding equivalent circuit, where Rs stands for the internal resistance of the electrolyte, Rct represents the resistance of charge transfer and the CPE is the constant phase element.

Table S3. The Li-ion transference number (t^+) of QSSCE with different content of LAGP

LAGP content (wt%)	Initial current I_0 (μA)	Steady current I_S (μA)	Initial Resistance R_0 (Ω)	Steady Resistance R_S (Ω)	Li-ion transference number t^+
8.68	23.71	11.35	160	168	0.36
18.35	54.37	30.02	62	76	0.47
30.69	24.87	16.36	179	186	0.52
37.35	18.50	12.55	251	263	0.54
48.38	16.23	12.55	237	247	0.69
60.33	56.82	46.48	82	91	0.76
66.85	24.60	22.01	342	377	0.83
78.83	10.67	8.11	440	483	0.66
87.74	12.11	10.38	693	750	0.62

*The Li-ion transference number (t^+) was calculated according to the following equation, where ΔV was 10mV:

$$t^+ = \frac{I_S(\Delta V - I_0 R_0)}{I_0(\Delta V - I_S R_S)}$$

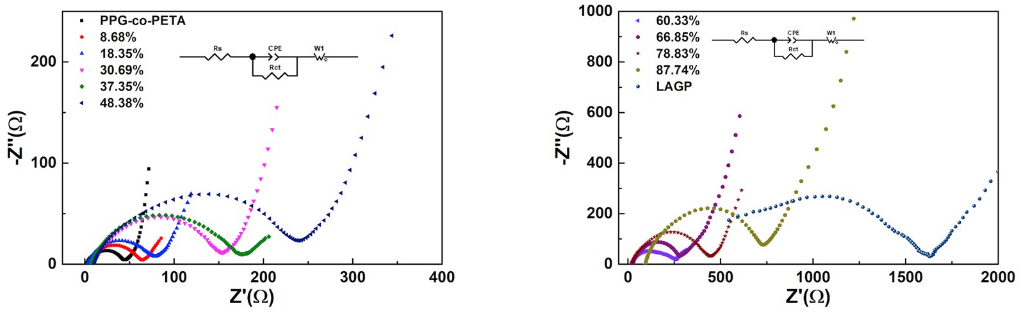

Figure S5. EIS curves of the QSSCE with different content of LAGP, inserted with the corresponding equivalent electrical circuit.

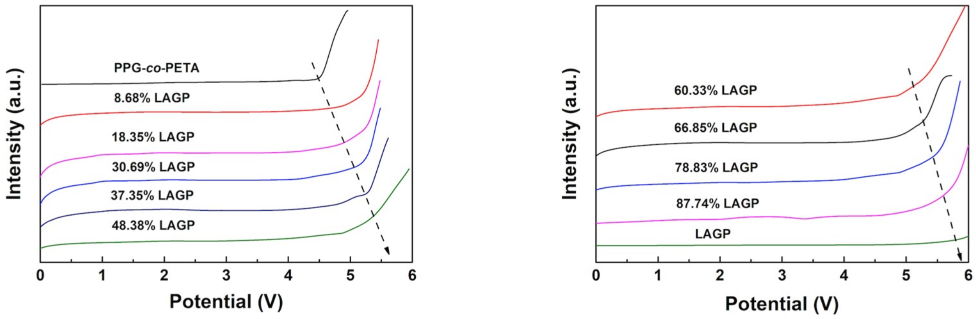

Figure S6. The electrochemical stability curves of all electrolytes measured by linear sweep voltammetry (LSV) method.
